# Increased serum levels of IL-40 are associated with IgA and NETosis biomarkers in Covid-19 patients: IL-40 and infectious diseases

**DOI:** 10.1371/journal.pone.0321578

**Published:** 2025-05-02

**Authors:** Umit Bagriacik, Resul Karakus, Melek Yaman, Nihan Oruklu, Milat Araz, Zafer Kalayci, Hasan Selcuk Ozger, Yesim Yildiz, Esin Senol

**Affiliations:** 1 Department of Immunology, Gazi University, Faculty of Medicine, Ankara, Türkiye; 2 Department of Infectious Diseases and Clinical Microbiology, Gazi University, Faculty of Medicine, Ankara, Türkiye; Central University of Tamil Nadu, INDIA

## Abstract

Interleukin 40 (IL-40) is a novel cytokine that has been associated with B lymphocytes, particularly IgA-secreting cells in gut mucosa. Considering mucosal association of IL-40, we aimed to determine serum IL-40 levels in patients with Covid-19. We compared IL-40 concentrations in healthy people to both patients with mild symptoms of SARS-CoV-2 infection and pneumonia. IL-40 was measured by ELISA. Serum IgA levels were tested by nephelometry. For the first time, we demonstrated that SARS-CoV-2 infection increased serum IL-40 levels significantly. The elevation of IL-40 in serum was related to severity of the infection. Therefore, IL-40 concentrations were quite higher in patients with symptoms of pneumonia. Our findings indicated that IgA and NETosis biomarkers were related with IL-40 increase. Based on these findings, we speculated that IL-40 would be associated with immune activities in the mucosa of the lungs of SARS-CoV-2-infected patients. This association may be linked to a mechanism that has a control on IgA and NETosis.

## Introduction

Interleukin 40 (IL-40) is a recently identified B cell-associated cytokine [1]. Only a few studies describing relations of IL-40 to autoimmune diseases have been published so far [1–5]. Therefore, involvement of IL-40 in many diseases are still unknown. Particularly, no publication exists to characterize serum levels of IL-40 in infected patients with SARS-CoV-2 or any other infection.

IL-40 is a 27 kDa-secreted protein that is unrelated to the other cytokine families. It is encoded by C17orf99 gene which is located on chromosome 17 in humans. Initially, it was reported that IL-40 was expressed in the bone marrow and fetal liver as well as in the periphery by activated B lymphocytes [1,2]. However, most recent studies indicated that activated neutrophils [3,4] and other sources [5] were also capable of producing IL-40. It was demonstrated that IgA levels in serum, feces, and milk reduced significantly in IL40 gene knock out (Il40^-/-^) mice while serum levels of IgM and IgG were not affected [1]. Based on this finding, it was concluded that IL-40 might be involved in development or control of IgA production.

Covid-19 is a recent pandemic which was caused by the infection of severe acute respiratory syndrome coronavirus 2 (SARS-CoV-2). More than seven million people died due to the infections of SARS-CoV-2. The virus enters host cells by binding to angiotensin-converting enzyme 2 (ACE2). Therefore, it infects not only the respiratory epithelial cells in the lung mucosa but almost many other cell types in every organ of the body. The massive viral infection initiates cellular perturbations and very sophisticated hyper-inflammatory reactions resulting in substantial amount of cytokine secretion or cytokine storm. The nature and the composition of the cytokine storm has been studied extensively by identifying many cytokines involvement [6,7]. Unfortunately, the significance of IL-40 in SARS-CoV-2 infections remains unknown.

NET formations were found out in 1996 by Takei et al [8] who displayed phorbol 12-myristate 13-acetate (PMA)-treated neutrophils exerted a distinct morphology from either apoptosis or necrosis. This process of NET formation named NETosis were described in detail by Brinkmann et al [9]. Major biomarkers for NETosis are degradative enzymes such as neutrophil elastase (NE), proteinase 3 (PR3) and myeloperoxidase (MPO) in granules of neutrophils [10]. Recent investigations revealed that NETosis has been correlated with severity and pathogenesis of Covid-19 [11,12]. However, any study for correlating NETosis with IL-40 during Covid-19 infection has been elucidated yet.

We aimed to investigate the functional relations between the serum levels of IL-40, IgA, and NETosis biomarkers in SARS-CoV-2 infections using frozen serum samples of patients who were hospitalized during the initial infections of the virus before vaccines came out.

## Materials and methods

### Patients and sample collection

A total of 60 patients diagnosed with SARS-Cov-2 infection and 30 healthy volunteers participated to the study. Half of the patients (30 patients) had mild symptoms while the other half (30 patients) had pneumonia (the criteria for mild COVID and pneumonia is explained in the result section). As soon as patients were admitted to the hospital, a 15-ml of blood was withdrawn in BD Vacutainer® Heparin Tubes. We used the cryopreserved serum samples from patients recruited between 15 June 2020 and 31 Dec 2021 during the first wave of the Covid-19 pandemic for a study of Covid-19. Therefore, the participating volunteers were not infected with SARS-CoV-2 in their previous life. They were not vaccinated with any kind of Covid-19 vaccine in their previous life either. The local ethics committee at Gazi University approved the use of cryopreserved serum samples. (Approval No: 1045, Approval Date: 13 Nov 2023). Participants were informed second time about reuse of their cryopreserved serum samples in the present study and the experimental procedures. The volunteers signed a new written consent form to confirm their participation to the second study. Based on this ethical permission, blood samples were accessed for research purposes in 15 January 2024 when we conducted ELISA assays. All experiments were conducted according to the rules in the Declaration of Helsinki.

A 15-ml of blood was withdrawn in BD Vacutainer® Tubes that were centrifuged in a centrifuge (Allegra^®^ X-12R, Beckman & Coulter, USA) at 2500 rpm for 15 minutes at room temperature. Serum samples were transferred to sterilized Eppendorf tubes that were preserved at minus 80 degree of Celsius in a freezer (Sanyo MDF-U76VC VIP+ Series Ultra-Low Temperature Freezer, Japan) until use.

### Enzyme-linked immunosorbent assay (ELISA)

We performed measurements for serum IL-40 (Abbexa, Cat. No: abx508530, Cambridge, United Kingdom) MPO (USCN, Cat. No: SEA601Hu, Wuhan, China), NE (Elabscience, Cat. No: E-EL-H1970 Houston, TX, USA), and PR3 (Elabscience, Cat. No: E-EL-H1946 Houston, TX, USA) by using commercial ELISA kits. ELISA was performed according to the instructions provided in the content of each ELISA kit. Briefly, serum samples were added to the wells of ELISA plates and incubated at room temperature for the time instructed in the test procedures by the vendor. After washing three times by a microplate washer (Tecan, HydroFlex™ plus, Switzerland), antibody conjugates were added in wells of microplate. Plates were incubated at room temperature for the time instructed in the test procedures by the vendor. Plates were washed three times by the microplate reader. Following an enzymatic reaction, absorbance value was measured at 450 nm by a plate reader (Biotek, Synergy HT, USA).

### Serum total IgA measurements

Serum IgA was measured by using a commercial kit (Beckman&Coulter, Cat No: 446460, Miami, FL, USA) in a nephelometer (Beckman&Coulter, Immage 800, Miami, FL, USA) according to the instructions provided by the vendor.

### Statistical analyses

Experimental data was analyzed by using a software, GraphPad Prism version 10 (GraphPad Software, Inc., San Diego, CA, USA) for statistical analyses. Statistical differences were determined using one way ANOVA for more than two groups. Paired or unpaired Student two-tailed t test (two groups) or a nonparametric Wilcoxon matched-pairs two-tailed test (matched samples) (Mann-Whitney Test) or Welch’s correction were used. Chi-square test was also used when necessary. X-Y correlation analysis with Pearson correlation coefficient was performed (95% confidence intervals). Value of p ≤ 0.05 was considered statistically significant.

## Results

### Demographic pathophysiologic features of patients

Patients were separated in two groups based on World Health Organization’s (WHO) guidance for clinical management of COVID-19 [13]. All patients were PCR positive for SARS-CoV-2. In the department of Infectious Diseases, physicians diagnosed pneumonia cases by evaluation of posteroanterior chest radiography or thoracic CT (computerized tomography scan). Patients with pneumonia were considered as severe COVID-19 if they had one of the following: oxygen saturation (SaO2) ≤ 90% or the ratio of partial pressure of oxygen to fraction of inspired oxygen (PaO2/FiO2) ≤ 300 or respiratory rate ≥ 30 per minute. COVID-19 patients without pneumonia and none of the above severity criteria were considered as mild cases. The first group included patients having mild symptoms of Covid-19, so we named them mild Covid while the second group included patients with heavy symptoms of pneumonia. There were significant differences in the number of lymphocytes, leukocytes, and neutrophils (Table 1).

**Table 1:** Demographical and clinical characteristics of participants.

Variables	Healthy (n:30)	Mild Covid (n: 30)	Pneumonia (n: 30)	p- value (< 0.05)*
Gender *n (%)*				
Male	17 (56.66)	11 (36.66)	17 (56.66)	–
Age *mean* *(min to max)*	41.27 (22–69)	39.83 (21–59)	49 (30–73)	0.0062*
Neutrophil, mm^3^ *(min to max)*	3.37 (1.8 to 5.1)	4.67 (1.99 to 9.54)	4.22 (2.06 to 7.55)	0.0018*
Leukocyte, mm^3^ *(min to max)*	5.13 (3.9 to 7.3)	7.42 (4.36 to 10.3)	6.08 (3.34 to 9.55)	<0.00001*
Lymphocyte, mm^3^ *(min to max)*	2.64 (1.3 to 4.1)	2.1 (1.2 to 3.4)	1.32 (0.40 to 2.66)	<0.00001*
NLR *(min to max)*	1.4 (0.7 to 2.92)	2.4 (1.08 to 5.08)	4.17 (1.23 to 14.37)	<0.00001*
CRP, mg/dL *(min to max)*	2.25 (1.0 to 4.1)	5 (1–43)	64 (2–209)	<0.00001*

CRP, C-reactive protein; NLR, Neutrophil Lymphocyte Ratio, One-Way Anova, (< 0.05)*

**Fig 1:**
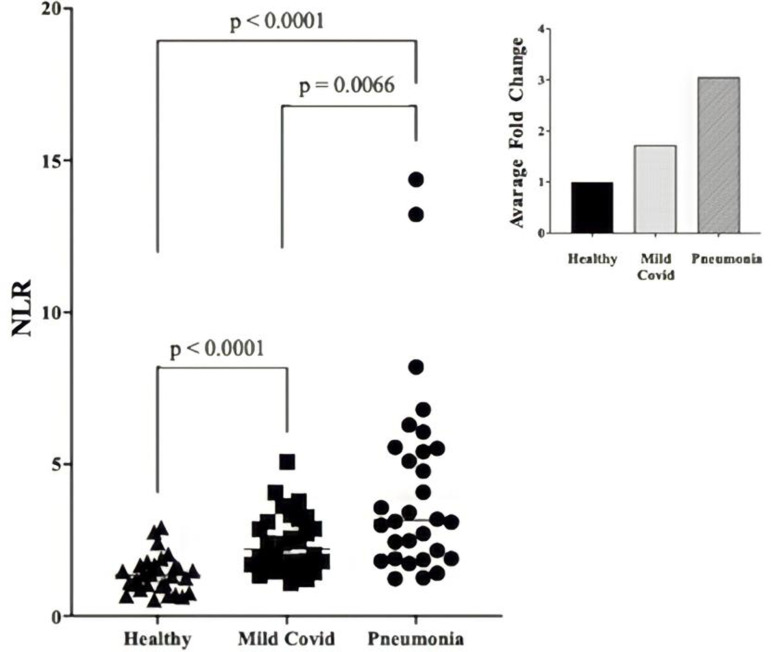
Comparison of NLR (Neutrophil/Lymphocyte Ratio) values. NLR values in healthy, mild covid and pneumonia patients were compared. NLR values increased significantly (p < 0.05) in patients in comparison to the healthy subjects. The increases were calculated as fold change that is shown on the right upper corner. Especially, NLR in pneumonia patients is three times of the NLR in healthy subjects.

The neutrophil-to-lymphocyte ratio (NLR) was described as a pathophysiological reflection of cellular immune response in adaptive and innate interactions during prolonged inflammation and used as an inflammatory biomarker in many diseases [14]. NLR values higher than 3.0 are pathologic and express broken balance between systemic inflammation and immunity [15]. NLR elevated significantly in pneumonia patients (Table 1). NLR was significantly higher in pneumonia patients comparing to that in either healthy people or mild covid patients (Fig 1). Especially, we detected a three-fold increase in pneumonia patients.

### Serum IL-40 values

**Fig 2:**
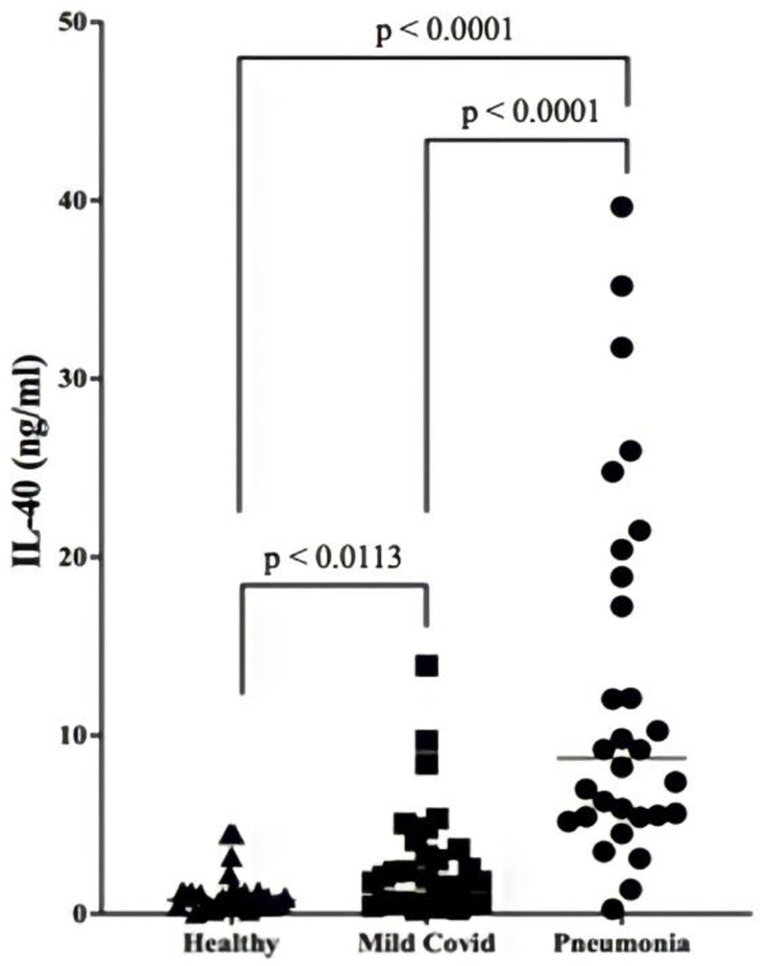
Serum IL-40 levels. Serum IL-40 levels in healthy subjects, in SARS-CoV-2 infected patients with mild symptoms and in SARS-CoV-2 infected patients with pneumonia were compared. The dramatic (p < 0.05) increase in pneumonia patients reflected the disease severity.

We first measured serum levels of IL-40 by ELISA in blood samples collected from patients who were diagnosed with Covid-19. IL-40 increased in both group of patients at different levels of significance in comparison to the healthy volunteers (Fig 2). The significance of the increase in mild covid patients was moderate (p = 0.0113) whereas the significance of increase in patients with pneumonia was so dramatic (p < 0.0001). Additionally, the difference between mild covid and pneumonia patients was significant as well (p < 0.0001). These findings indicated that serum levels of IL-40 were associated with severity of the infection.

### Serum IgA values

**Fig 3:**
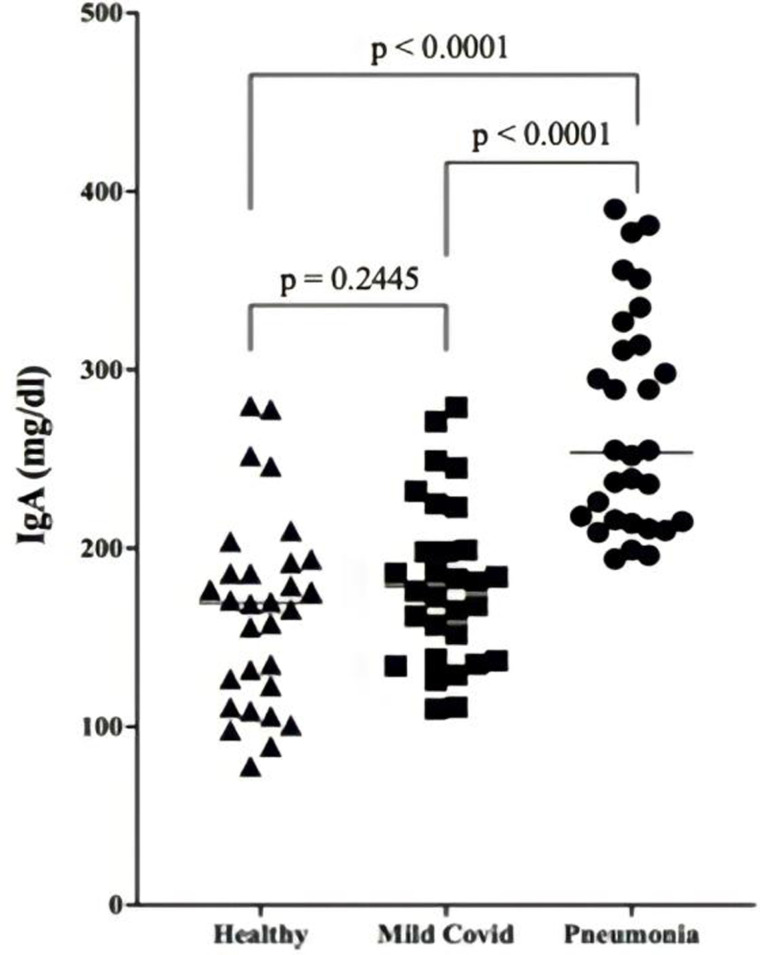
Serum total IgA levels. Serum IgA levels in healthy donors, in SARS-CoV-2 infected patients with mild symptoms and in SARS-CoV-2 infected patients with pneumonia were compared. The elevation in pneumonia patients was significant (p < 0.05).

A recent study indicated that IL-40 deficiency could be functionally related to lower IgA production in mucosal sites [2]. Covid-19 is an infection of lung mucosa. Therefore, we determined serum levels of IgA to reach any clinical relation between IL-40 and IgA production in patients with Covid-19. Although there was a slight elevation in IgA in mild covid patients, it was not significant at all (Fig 3). However, serum IgA increased significantly (p < 0.0001) in patients with pneumonia. This increase was related to severity of the infection.

### Correlation between IL-40 and IgA

**Fig 4:**
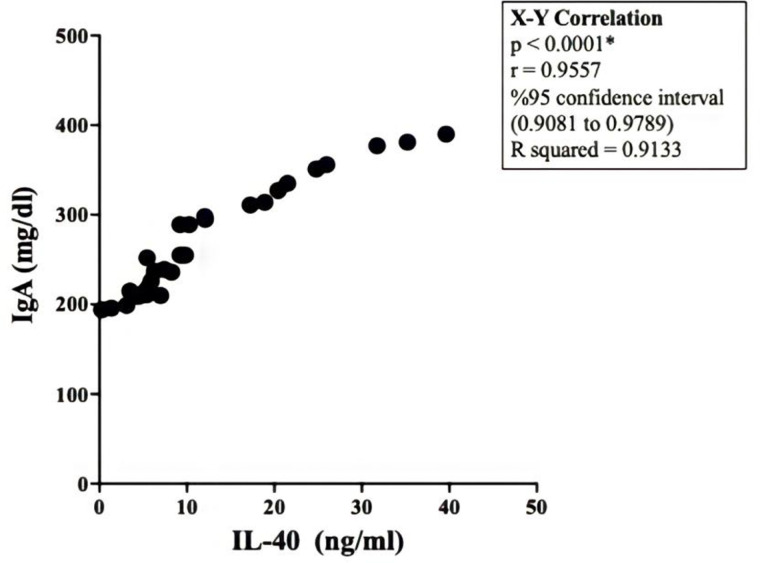
Correlation between IL-40 and IgA. X-Y correlation was calculated by Graphpad Prism 10 software at 95% confidence interval. A significant correlation between IL-40 and IgA in pneumonia patients was remarkable. This correlation indicated that acceleration in both IL-40 and IgA was related to the disease severity.

Since a dramatic increase in both IL-40 and IgA in pneumonia patients was detected, we determined a possible correlation between IL-40 and IgA. X-Y correlation was calculated at 95% confidence interval. A significant (p < 0.0001) correlation was recorded (Fig 4). This finding indicated that both IL-40 and IgA was related to the severity of infection under a correlated mechanism which could be related to an important immune regulation.

### NETosis biomarkers

**Fig 5:**
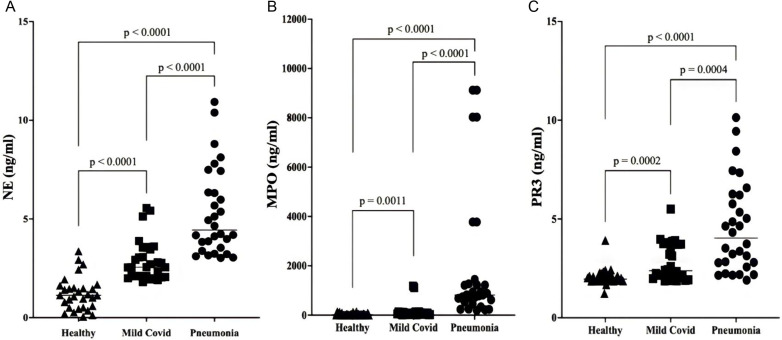
Serum NETosis biomarkers. Serum NETosis biomarker levels in healthy donors, in SARS-CoV-2 infected patients with mild symptoms and in SARS-CoV-2 infected patients with pneumonia were compared. **(A)** Serum myeloperoxidase (MPO) levels, **(B)** Serum neutrophil elastase (NE) levels, and **(C)** serum proteinase 3 (PR3) levels. (p < 0.05).

MPO, PR3, and NE are Netosis-associated biomarkers. We determined serum levels of those biomarkers. All the markers increased in both mild covid and pneumonia patients significantly (Fig 5). The most dramatic escalation was in NE (Fig 5A). We observed similar raise for MPO (Fig 5B) and PR3 (Fig 5C) as well. The magnitude of increase in pneumonia patients was quite higher. There was a significant difference (p < 0.0001) between mild covid and pneumonia for all the markers indicating that NETosis was quite severe in patients with pneumonia comparing with patients with mild symptoms (Fig 5A–5C).

**Fig 6:**
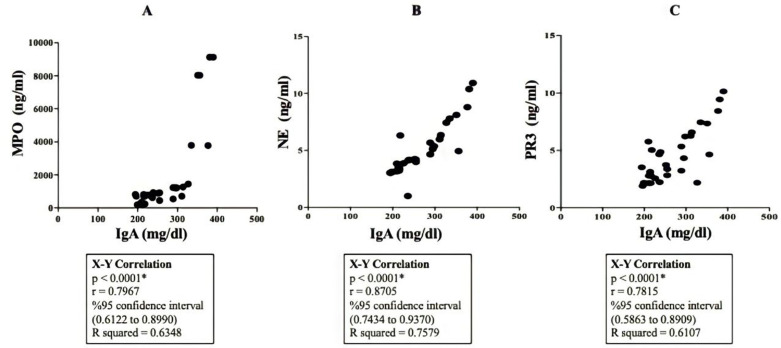
Correlation between IgA and NETosis-associated biomarkers. X-Y correlation was calculated by Graphpad Prism 10 software at 95% confidence interval. Significant correlations between IgA and NETosis-associated biomarkers in pneumonia patients were observed. **(A)** Correlation between IgA and myeloperoxidase (MPO), **(B)** Correlation between IgA and neutrophil elastase (NE), and **(C)** Correlation between IgA and proteinase 3 (PR3). (p < 0.05).

A simultaneous increase in IgA and NETosis-associated biomarkers in pneumonia patients indicated a possible correlation between them. Therefore, we performed statistical analyses at 95% confidence interval. In fact, we found significant (p < 0.0001) correlations between IgA and all those NETosis biomarkers (Fig 6A–6C). Those correlation were related to the disease severity as well.

## Discussion

This study particularly focused on the association between serum levels of IL-40 and IgA in SARS-CoV-2-infected patients. Also, we expanded the study to NET formation and severity of infection. By doing so, for the first time, we demonstrated that IL-40 levels may be in association with the severity of infection. This may involve IgA as well as NETosis biomarkers.

IL-40 is a recently identified orphan cytokine. Latest studies suggested that IL-40 was involved in autoimmune diseases such as primary Sjogren’s syndrome [5], ankylosing spondylitis [16], rheumatoid arthritis [3], systemic lupus erythematosus [17], and type 2 diabetes [18]. Studies illustrated that IL-40 was related to the disease activity in autoimmune diseases. For example, IL-40 elevated during early and active stages of rheumatoid arthritis, primary Sjögren’s syndrome and systemic lupus erythematosus [4,5,17].

We determined that IL-40 and IgA in serum of pneumonia patients increased significantly. This finding indicated a relation between serum levels of IL-40 and IgA. The present study is the first one demonstrating involvement of IL-40 in acute phases of inflammatory reactions in an infectious disease. IL-40 deficient mice exerted reduced levels of IgA in the serum, gut, feces, and lactating mammary gland [1] implying that IL-40 exerted a positive regulation on IgA levels. In fact, we observed a significant correlation between serum IL-40 and IgA levels. This finding may indicate a possible regulatory mechanism by IL-40 on IgA levels. This speculation could be expanded that the regulation may be either towards to increases in IgA synthesis itself or towards to increases in the number of B cells secreting IgA. However, currently, we do not know existence of such a mechanism yet.

IgA plays an essential role during COVID-19 infection by neutralizing the virus at mucosal sites to reduce its transmission [19]. We observed a non-significant elevation in serum total IgA of mild covid patients. However, IgA increased strongly in patients with pneumonia. This finding suggested that serum IgA is associated with severity of the disease. Zervou et al reported that IgA was the predominant immunoglobulin in early periods of Covid-19 infections regardless of age and sex [20]. They showed that IgA levels did not change significantly in patients having mild and moderate disease. However, IgA levels increased significantly in patients having severe and critical disease. Therefore, they correlated IgA with severity of the disease. Several other studies reported similar results confirming that serum IgA levels was related to the severity of Covid-19 infection [21,22]. Increase in serum IgA levels was associated with increase in serum levels of TGF-β1 which is a switch factor for IgA isotype of immunoglobulins [23]. In fact, it was suggested that TGF-β1 was responsible for abnormal IgA responses and fatal symptoms of pulmonary fibrosis observed during severe Covid-19 infections [23]. All together those published findings are directly consequent to our findings about the association of IgA with the disease severity.

Irregular cytokine release results in uncontrolled hyperinflammation that is called cytokine storm syndrome in COVID-19 patients. Therefore, cytokine and chemokine homeostasis are very crucial in development of pathogenesis during the disease. We and others demonstrated that several cytokines and chemokines including IL‐2, IL‐6, IL‐7, IL‐10, IL-15, IL-17, IP‐10, MCP‐1, TNF‐α, GCSF were related to disease severity and mortality in COVID-19 [2,24]. Except for IgA, currently, we do not have any descriptive evidence for a mechanism by which IL-40 is involved in any regulatory events in COVID-19. The positive correlation between IL-40 and IgA is suggesting that IL-40 levels is affecting IgA levels. However, it is too early to suggest that this positive correlation would be beneficial or harmful during the severe infections of COVID-19. Further mucosal studies would reveal the scene behind those possible mechanisms.

We demonstrated a significant change in NETosis biomarkers. Especially we observed dramatic elevation in MPO, NE and PR3. In fact, numerous studies have published NETosis biomarkers increased during SARS-CoV-2 infection [11,12,25,26]. Several investigations described NETosis biomarkers as a predictor of disease severity [25,26]. Our results supported this common notion that NETosis biomarkers increased depending on the severity of Covid-19 infection. Especially, MPO, NE and PR3 raised more severely in patients with pneumonia comparing to the patients with mild symptoms. Therefore, MPO, NE and PR3 were the clinical biomarkers showing obvious relations with disease severity.

It was reported that IgA enhanced NETosis via Fcα receptor I (FcαRI) in response to IgA-opsonized particles or IgA-opsonized bacteria [27]. Another study confirmed the effect of IgA on NETosis induction [28]. Additionally, a recent study revealed that IgA potentiated NETosis in response to viral infections [29]. Those studies suggested IgA-immune complexes stimulated suicidal NETosis which was independent of toll-like receptor signaling. This process involved activation of NE, MPO, nicotinamide adenine dinucleotide phosphate (NADPH) oxidase and production of reactive oxygen species (ROS). We demonstrated serum levels of NETosis biomarkers such as NE, MPO, PR3 increased simultaneously with serum IgA levels. This may suggest that IgA could have a functional relation with increased NETosis processes during the infection periods in patients with pneumonia.

Based on our findings, we speculated that IL-40 may up-regulate IgA in mucosal sites during infections. This upregulation may require a certain plateau of IL-40 concentration. IgA did not increase significantly in patients with mild symptoms comparing to the healthy volunteers as IL-40 increase was not robust either. So, the increase in IL-40 was sufficient to trigger IgA upregulation. On the other hand, IgA raise was quite significant in patients with pneumonia as the magnitude of IL-40 acceleration was predominant. This positive association between IL-40 and IgA may be involved in some regulatory effects via FcαRI on production of NETosis biomarkers.

One can argue that we did not demonstrate NET formation by classical histological staining of extracellular DNA. After activation of neutrophils, NETosis biomarkers such as NE, peptidyl-arginine deaminase 4 (PAD4), MPO and PR3 migrate to the nucleus and accumulate there during NET formation [10]. Eventually those biomarkers of NET formation are released into circulation. So, they can be determined as reflection of NETosis in serum during human diseases. There are several tools for assessment of measuring NET formation. ELISA is a common method in quantifying NETosis and very useful by associating NETosis biomarkers with clinical conditions of human diseases [30]. Numerous studies used ELISA to quantify serum biomarkers of NETosis in human diseases [31,32]. Therefore, we assessed circulating NETosis biomarkers by using ELISA.

Taken together, we reported for the first time that IL-40 increased significantly in serum of patients with SARS-CoV-2 infection. This increase was correlated with IgA and NETosis-associated biomarkers. We observed a robust increase in patients with pneumonia comparing to the patients with a mild infection suggesting that the increase in serum levels of IL-40 was related to the severity of infection. These finding are implying that IL-40 is also involved in some immune mechanisms in infectious diseases as well as in autoimmune diseases. Those immune mechanisms include at least IgA and NETosis biomarkers and requires further characterization.

## Conclusion

In conclusion,we identified that IL-40 was associated with COVID-19, an infectious disease. In fact, serum IL-40 levels were related to severity of SARS-CoV-2 infection. The mechanism underlying how IL-40 exerts its immunologic effects is still unknown. However, our study indicated a possible relation to IgA and NETosis pathways. Identification of IL-40-mediated immune mechanisms would shed a light on discoveries of new therapeutic drug targets for infectious diseases as well as autoimmune diseases.

## Supporting information

S1 File CRP DATA. (PDF)

S2 File IgA DATA. (PDF)

S3 File IL-40 DATA. (PDF)

S4 File MPO DATA. (PDF)

S5 File NE DATA. (PDF)

S6 File NLR DATA. (PDF)

S7 File PR3 DATA. (PDF)
