# Supplementary material for: Increased serum levels of IL-40 are associated with IgA and NETosis biomarkers in Covid-19 patients: IL-40 and infectious diseases
Source: PLoS One. 2025 May 2;20(5):e0321578. doi: 10.1371/journal.pone.0321578 (PMC12047749; doi:10.1371/journal.pone.0321578)
Supplement: S1 File — (PDF) [file pone.0321578.s001.pdf]

LEUKOCYTE AND CRP VALUES

Individual No	Healthy		Mild Covid		Pneumonia	
	Leukocyte (mm^3)	CRP (mg/dL)	Leukocyte (mm^3)	CRP (mg/dL)	Leukocyte (mm^3)	CRP (mg/dL)
**1**	4,9	2,5	10,3	1,36	6,11	6,32
**2**	5,2	1,1	7,87	4,67	5,61	8
**3**	[redacted]					
**4**						
**5**						
**6**						
**7**						
**8**						
**9**						
**10**						
**11**						
**12**						
**13**						
**14**						
**15**						
**16**						
**17**						
**18**						
**19**						
**20**						
**21**						
**22**						
**23**						
**24**						
**25**						
**26**						
**27**						
**28**						
**29**	5,1	1,3	6,64	5,57	7,424	8,38
**30**	4,8	3,6	5,19	4,43	5,45	64,4
**Average**	**5,13**	**2,25**	**7,42**	**4,54**	**6,10**	**64,07**
**SD**	**1,00**	**0,94**	**1,87**	**7,48**	**1,74**	**63,11**
