# Supplementary material for: Increased serum levels of IL-40 are associated with IgA and NETosis biomarkers in Covid-19 patients: IL-40 and infectious diseases
Source: PLoS One. 2025 May 2;20(5):e0321578. doi: 10.1371/journal.pone.0321578 (PMC12047749; doi:10.1371/journal.pone.0321578)
Supplement: S2 File — (PDF) [file pone.0321578.s002.pdf]

IgA VALUES and ANALYSES

PATIENT NO	HEALTHY CONTROL	MILD COVID	PNEUMONIA
	IgA mg/dL		
1	78	172,8	218
2	135	232	356
[redacted]			
29	176	198	289
30	156	165	236
Average	165,31	180,39	269,83
SD	53,63	45,39	61,63
