# Supplementary material for: Increased serum levels of IL-40 are associated with IgA and NETosis biomarkers in Covid-19 patients: IL-40 and infectious diseases
Source: PLoS One. 2025 May 2;20(5):e0321578. doi: 10.1371/journal.pone.0321578 (PMC12047749; doi:10.1371/journal.pone.0321578)
Supplement: S3 File — (PDF) [file pone.0321578.s003.pdf]

IL-40 VALUES AND ANALYSES

PATIENT NO	HEALTHY CONTROL	MILD COVID	PNEUMONIA
	IL-40 ng/ml		
1	1,22	0,448	5,523
2	4,499	5,064	25,962
[redacted]			
29	2,237	3,256	10,256
30	0,987	2,091	8,231
Average	1,11	2,82	12,42
SD	1,12	3,14	10,35
