# Supplementary material for: Increased serum levels of IL-40 are associated with IgA and NETosis biomarkers in Covid-19 patients: IL-40 and infectious diseases
Source: PLoS One. 2025 May 2;20(5):e0321578. doi: 10.1371/journal.pone.0321578 (PMC12047749; doi:10.1371/journal.pone.0321578)
Supplement: S4 File — (PDF) [file pone.0321578.s004.pdf]

# MPO VALUES AND ANALYSES (ng/ml)

| PATIENT NO | HEALTHY CONTROL | MILD COVID | PNEUMONIA |
|---|---|---|---|
| | MPO ng/ml | | |
| 1 | 25,5 | 75,5 | 236 |
| 2 | 143,5 | 129,5 | 8028 |
| 3 | [redacted] | | |
| 4 | | | |
| 5 | | | |
| 6 | | | |
| 7 | | | |
| 8 | | | |
| 9 | | | |
| 10 | | | |
| 11 | | | |
| 12 | | | |
| 13 | | | |
| 14 | | | |
| 15 | | | |
| 16 | | | |
| 17 | | | |
| 18 | | | |
| 19 | | | |
| 20 | | | |
| 21 | | | |
| 22 | | | |
| 23 | | | |
| 24 | | | |
| 25 | | | |
| 26 | | | |
| 27 | | | |
| 28 | | | |
| 29 | 4,4 | 87,6 | 1232 |
| 30 | 8 | 50,4 | 742 |
| **Average** | **40,44** | **173,33** | **1977,77** |
| **SD** | **44,25** | **426,30** | **2770,25** |
