# Supplementary material for: Increased serum levels of IL-40 are associated with IgA and NETosis biomarkers in Covid-19 patients: IL-40 and infectious diseases
Source: PLoS One. 2025 May 2;20(5):e0321578. doi: 10.1371/journal.pone.0321578 (PMC12047749; doi:10.1371/journal.pone.0321578)
Supplement: S5 File — (PDF) [file pone.0321578.s005.pdf]

NE VALUES AND ANALYSES

PATIENT NO	HEALTHY CONTROL	ASYMPTHOMATIC	PNEUMONIA
	NE ng/ml		
1	0,7871	2,549	6,313
2	1,178	3,051	4,936
3	[redacted]		
4	[redacted]		
5	[redacted]		
6	[redacted]		
7	[redacted]		
8	[redacted]		
9	[redacted]		
10	[redacted]		
11	[redacted]		
12	[redacted]		
13	[redacted]		
14	[redacted]		
15	[redacted]		
16	[redacted]		
17	[redacted]		
18	[redacted]		
19	[redacted]		
20	[redacted]		
21	[redacted]		
22	[redacted]		
23	[redacted]		
24	[redacted]		
25	[redacted]		
26	[redacted]		
27	[redacted]		
28	[redacted]		
29	1,678	1,998	5,678
30	17,24	2,091	0,987
**Average**	**1,74**	**2,87**	**5,10**
**SD**	**3,04**	**1,03**	**2,31**
