# Supplementary material for: Increased serum levels of IL-40 are associated with IgA and NETosis biomarkers in Covid-19 patients: IL-40 and infectious diseases
Source: PLoS One. 2025 May 2;20(5):e0321578. doi: 10.1371/journal.pone.0321578 (PMC12047749; doi:10.1371/journal.pone.0321578)
Supplement: S7 File — (PDF) [file pone.0321578.s007.pdf]

PR3 VALUES AND ANALYSES

PATIENT NO	HEALTHY CONTROL	MILD COVID	PNEUMONIA
	PR3 ng/ml		
1	1,856	3,81	5,032
2	2,007	1,982	4,64
3	[redacted]		
4			
5			
6			
7			
8			
9			
10			
11			
12			
13			
14			
15			
16			
17			
18			
19			
20			
21			
22			
23			
24			
25			
26			
27			
28			
29	1,678	2,334	3,231
30	2,432	3,456	4,654
**Average**	**2,07**	**2,81**	**4,58**
**SD**	**0,42**	**0,93**	**2,30**
