# Supplementary material for: Increased serum levels of IL-40 are associated with IgA and NETosis biomarkers in Covid-19 patients: IL-40 and infectious diseases
Source: PLoS One. 2025 May 2;20(5):e0321578. doi: 10.1371/journal.pone.0321578 (PMC12047749; doi:10.1371/journal.pone.0321578)
Supplement: S6 File — (PDF) [file pone.0321578.s006.pdf]

NEUTROPHIL / LYMPHOCYTE RATIO (NLR)

No	Healthy		Mild Covid		Pnemonia		NLR		
	Lymphocyte (mm^3)	Neutrophil (mm^3)	Lymphocyte (mm^3)	Neutrophil (mm^3)	Lymphocyte (mm^3)	Neutrophil (mm^3)	Healthy	Mild Covid	Pneumonia
1	2,2	3,3	10,3	6,39	6,11	3,75	1,50	2,56	2,16
2	2,1	2,2	7,87	5,31	5,61	2,69	1,05	2,41	1,23
3	[redacted]								
4									
5									
6									
7									
8									
9									
10									
11									
12									
13									
14									
15									
16									
17									
18									
19									
20									
21									
22									
23									
24									
25									
26									
27									
28									
29	3,1	3,9	6,64	4,74	0,41	0,01	1,26	3,62	5,56
30	2,3	4,7	5,19	3,11	3,34	3,28	2,04	1,80	6,8
**Average**	**2,67**	**3,37**	**7,42**	**4,67**	**5,70**	**3,94**	**1,39**	**2,40**	**4,25**
**SD**	**0,96**	**1,01**	**1,87**	**1,53**	**2,24**	**1,88**	**0,60**	**0,96**	**3,18**

Fold Change in NLR	Healthy	Mild Covid	Pneumonia
	**1**	**1,73**	**3,06**
